# VDR Polymorphic Variants Are Related to Improvements in CRP and Disease Activity in Patients with Axial Spondyloarthritis That Undergo Anti-TNF Treatment

**DOI:** 10.3390/genes13101873

**Published:** 2022-10-16

**Authors:** Bartosz Bugaj, Joanna Wielińska, Jerzy Świerkot, Katarzyna Bogunia-Kubik, Katarzyna Górna

**Affiliations:** 1 Department of Rheumatology and Internal Medicine, Wroclaw Medical University, 50-556 Wroclaw, Poland; 2 Laboratory of Clinical Immunogenetics and Pharmacogenetics, Hirszfeld Institute of Immunology and Experimental Therapy, Polish Academy of Sciences, 53-114 Wroclaw, Poland

**Keywords:** axial spondyloarthritis (axSpA), ankylosing spondylitis (AS), vitamin D receptor (VDR), single nucleotide polymorphism (SNP), disease progression, anti-TNF therapy

## Abstract

Vitamin D deficiency is related with susceptibility or progression of various autoimmune diseases. The aim of the study was to assess potential relations between single nucleotide polymorphisms (SNPs) in the vitamin D receptor-coding gene (*VDR*): rs1544410 (*Bsm*I), rs2228570 (*Fok*I), rs731236 (*Taq*I), rs7975232 (*Apa*I), and disease activity in patients with axial spondyloarthritis (axSpA) undergoing anti-TNF therapy. The *VDR* rs731236 *CT* genotype was statistically more common among female patients (*p* = 0.027). An improvement of CRP equal to or higher than 50% after 3 months of anti-TNF therapy was observed for rs2228570 *T* allele (*p* = 0.002). After 6 months, CRP improvement equal to or higher than 75% was related to presence of the rs1544410 *AA* genotype (*p* = 0.027) and the rs731236 *CC* homozygotes (*p* = 0.047). Baseline BASDAI values were lower in individuals with the rs2228570 *TT* genotype (*p* = 0.036) and rs7975232 *C* allele (*p* = 0.029). After 6 months of treatment, lower BASDAI values were observed in *AC* heterozygotes (*p* = 0.005). The same *AC* genotype was more frequently detected in patients with remission (BASDAI ≤ 2) (*p* = 0.001) and in those achieving BASDAI improvement equal to or higher than 75% (*p* = 0.006). In conclusion, *VDR* SNPs were found to relate to CRP and BASDAI values at different time points of anti-TNF therapy.

## 1. Introduction

Vitamin D is a steroid hormone originating from cholesterol. Few foods naturally contain vitamin D, which is mostly synthesized in human skin through sun exposure. The biologically inactive precursor is converted in the liver and kidney to the biologically active form: 1,25-dihydroxy vitamin D (1,25(OH)2D), also known as calcitriol. The 1,25(OH)2D enters target cells and binds to the vitamin D receptor (VDR). This intracellular polypeptide acts as VDR/VDR homodimers or VDR/retinoic acid receptor (RXR) heterodimers (VDR/RXR) to target cell DNA, leading to special protein syntheses [1].

The primary function of 1,25(OH)2D is to regulate calcium and phosphorus homeostasis via actions in the intestines, kidneys, and bone [2,3]. Moreover, vitamin D signaling has an effect on many physiologic processes, including normal and malignant cell growth and differentiation, the innate and adaptive immune function, improved cardiovascular function, diabetes prevention, and the complex interplay with other hormones [4,5]. As for immunomodulatory actions of 1,25(OH)2D, it enhances the innate immune system and inhibits adaptative immune responses, which is associated with increased synthesis of interleukins by T helper (Th)-2 lymphocytes and the upregulation of regulatory T lymphocytes (T-reg) [6]. Vitamin D has also been reported to inhibit expression of TNF-α [7]. Clinical studies have indicated that vitamin D deficiency is positively correlated with the onset or exacerbation of various autoimmune diseases, such as type I diabetes mellitus, autoimmune thyroid diseases, multiple sclerosis, autoimmune rheumatic diseases, inflammatory bowel diseases, and psoriasis [8,9,10]. Experiments seeking to define the mechanisms underlying this finding are ongoing [11].

*VDR* gene polymorphisms may lead to functional changes reducing vitamin D regulatory effects on the immune response. Some associations have been identified with the onset of specific immune-mediated diseases: rs1544410 or rs731236 single nucleotide polymorphisms (SNPs) with autoimmune thyroid disease, rs1544410 and rs2228570 with systemic lupus erythematosus (SLE), rs2228570 with diabetic nephropathy, and finally, rs7975232, rs1544410, and rs731236 with rheumatoid arthritis (RA) [12]. A meta-analysis of studies confirmed the correlation of rs1544410 and rs731236 for RA patients, especially among Asians [13]. Additionally, the association of the *VDR* rs1544410 polymorphism with RA activity is interesting, as described by Gómez-Vaquero et al. [14]. High disease activity with low vitamin D levels has been described more extensively [9,15,16].

The findings on the impact of *VDR* polymorphisms on various rheumatic disorders encouraged us to investigate these potential relationships in another chronic inflammatory disease: axial spondyloarthritis (axSpA).

## 2. Materials and Methods

### 2.1. Participants

The study group consisted of 106 patients of Caucasian origin diagnosed with axial spondyloarthritis (axSpA) at Department of Rheumatology and Internal Medicine of Wrocław Medical University, Poland. The female to male ratio among the patients was 28:78. The study group included 86 ankylosing spondylitis (AS) patients and 20 persons with non-radiographic axSpA (nr-axSpA). AS patients were diagnosed according to modified New York criteria [17], while those with nr-axSpA were diagnosed using ASAS criteria [18]. All of the participants gave their informed consent regarding the study. Patients were excluded from the study according to the following criteria: age < 18 years, pregnancy or breastfeeding, cancer, chronic disease, organ failure exacerbation, mental retardation, and alcohol or drug abuse.

Clinical data of the qualified participants, such as age, body mass index (BMI), disease onset, peripheral arthritis history, and medication history were collected (Table 1) and used for downstream statistical analysis. In addition to musculoskeletal symptoms, extra-articular manifestations (such as uveitis, psoriasis, or inflammatory bowel disease) were also noted. All participants received one of the following anti-TNF treatments: adalimumab, certolizumab, etanercept, golimumab, or infliximab. After two nonsteroidal anti-inflammatory drugs (NSAIDs) were found to be ineffective or when contraindications to such treatment were present, then anti-TNF treatment was implemented. This marked the beginning of the participants’ observation for the current study. Apart from the mentioned baseline (day 0), the examination took place also after 12 weeks (3 months) and 24 weeks (6 months) of the anti-TNF therapy. Parameters, such as C-reactive protein (CRP) measured using the nephelometric method, erythrocyte sedimentation rate (ESR), and human leukocyte antigen B27 (HLA-B27) status, were assessed at each of the above-mentioned time points. CRP and ESR are non-specific inflammatory biomarkers often used to monitor the rheumatic diseases activity [19]. The first increases a few hours after the onset of inflammation or injury and peaks at 36 to 50 h. CRP levels drop quickly after inflammation resolves. ESR remains elevated for days to weeks after resolution of inflammation [20]. HLA-B27 status is a relevant tool that allows to distinguish SpA from no SpA, since it is strongly associated with SpA. This has been used in the axSpA ASAS criteria [21]. Back pain was quantified using the visual analogue scale (VAS) (range 0–100 mm). Disease activity was determined according to Bath Ankylosing Spondylitis Disease Activity Index (BASDAI) (range 0–10) [22]. It consists of 6 questions concerning the intensity of back pain and peripheral joint pain, morning stiffness, and the level of fatigue. Both BASDAI and VAS scores are completed by the patient. The initial axSpA activity at the beginning of the study was high and above 4 in BASDAI score.

As for the control group, 122 healthy donors for allogeneic haematopoietic stem cell transplantation were recruited. They were not diagnosed with any rheumatic or autoimmune disorder, nor with any other disease.

The study was conducted in accordance with the Declaration of Helsinki and approved by the Ethics Committee of the Wrocław Medical University (Wrocław, Poland) (protocol code: KB-751/2018, date of approval: 18 December 2018).

### 2.2. SNP Selection

Single nucleotide polymorphisms (SNP) in *VDR* gene were selected for the analysis based on the literature data and information available in the NCBI SNP database. Potential clinical or biological impact of the polymorphism (e.g., change in RNA and/or amino acid sequence, potential splicing site and/or miRNA binding site), as well as minor allele frequency (MAF, 1000 Genomes Project) above 10% in the European population were the most important factors when selecting SNPs for the study.

Four polymorphic variants in the *VDR* gene were selected for the experiment: rs2228570, rs1544410, rs7975232, and rs731236. The rs2228570 polymorphism (often called *Fok*I after the restriction enzyme widely used for its genotyping [23] is located in exon 1 of the *VDR* gene. It is a thymine (T) to cytosine (C) substitution that leads to changing methionine (Met) codon (ATG) into one that codes threonine (Thr) (ACG). As the mentioned Met marks the original start of translation, the variant allele introduces a new start codon: at methionine localized three codons downstream from the sequence. Consequently, the rs2228570 *T* variant generates VDR protein that is 3 amino acids shorter than the original one (the mutant with 424 codons vs. the wild type having 427 codons) [24]. Importantly, this short VDR isoform was reported as 1.7-fold more active than the longer one [25].

The second polymorphism selected for the study was rs1544410 (also called *Bsm*I). It is a guanine (G) to adenine (A) substitution in the intron region of the *VDR* gene. This genetic variant is hypothesized to affect mRNA stability and generate an alteration in splice site or a change in intron regulatory elements [26].

The two last SNPs included in the experiment were rs7975232 (*Apa*I) and rs731236 (*Taq*I). Those two are localized in a very close proximity in the *VDR* gene. As a consequence, usually the exact same primer sets are used for the PCR analysis of them both [23,27]. The rs7975232 cytosine (C) to adenine (A) substitution is localized in the intron and expected to affect mRNA stability and gene expression [28]. Meanwhile, the rs731236 SNP is located on a following exon. It is a thymine (T) to cytosine (C) substitution, leading to a silent mutation where one isoleucine (Ile) codon (ATT) is replaced by another isoleucine codon (ATC) in the sequence. Consequently, no change is observed in the amino acid chain of the VDR protein (based on the NCBI SNP database, as of 15 August 2022: https://www.ncbi.nlm.nih.gov/snp/rs731236#variant_details). However, this SNP could potentially have an effect on mRNA stability or modify the binding with vitamin D responsive elements (VDREs) located in the target genes [29,30].

### 2.3. Sample Collection and Genotyping

Peripheral blood of participants was collected into BD Vacutainer K2EDTA tubes (Becton, Dickinson, Franklin Lakes, NJ, USA) and stored frozen at −20 °C for later nucleic acid extraction. After storage, samples were thawed and brought to room temperature. Then, DNA was extracted using QIAmp DNA Blood Mini/Midi Kit (Qiagen, Hilden, Germany) according to the manufacturer‘s protocol.

SNP genotyping was performed using LightSNiP assays (TIB MOLBIOL, Berlin, Germany) and LightCycler FastStart DNA Master HybProbe (Roche Diagnostics GmbH, Mannheim, Germany) on the LightCycler 480 II Real-Time PCR Instrument (Roche Diagnostics GmbH, Basel, Switzerland).

Genotyping was performed according to the manufacturer‘s instructions. SNPs were detected by PCR amplification and labeling with specific probes. The following melting curve analysis provided visual means for distinguishing the wild type and variant alleles, as well as heterozygotes.

### 2.4. Statistical Analysis

Data and clinical parameters collected during experiments (listed in Table 1, as well as in Supplementary Tables) were analyzed statistically with the R software version 4.0.3 (http://www.rproject.org Assessed on 13 June 2022) and GraphPad Prism 7 for Windows (GraphPad Software, La Jolla, CA, USA). The GraphPad Prism 7 was also used to prepare the figures.

Data normality was verified with the Shapiro–Wilk test. Obtained results are presented as medians with interquartile ranges (IQRs) for quantitative data, or as numbers with percentages for categorical variables. Either the chi-square or Fisher‘s exact test was applied to compare genotypes and allele frequencies between the patient and control groups. The unpaired two-sample Wilcoxon test was introduced to identify associations between clinical parameters and genetic variants. To analyze relationships between categorical data and genotypes, Fisher’s exact test was used.

Genetics models were used to describe associations within investigated SNPs. The genotypes were coded as a major allele homozygote (MM), a heterozygote (Mm), and a minor allele homozygote (mm). A dominant model compared MM versus Mm + mm, whereas a recessive model compared MM + Mm versus mm. An over-dominant model was described as MM + mm versus Mm. The comparisons between MM and Mm, and mm were included into a codominant model.

Probability (*p*) values of 0.05 or lower (*p* ≤ 0.05) were considered statistically significant for all performed analyses.

Hardy–Weinberg equilibrium (HWE) was calculated manually using Microsoft Excel 2019 (version 16.0.10369.20032, Microsoft Corporation, Redmont, WA, USA).

Linkage disequilibrium between different VDR polymorphic variants was analyzed with Haploview 4.2 software. 

## 3. Results

### 3.1. Linkage Disequilibrium and Hardy-Weinberg Equilibrium (HWE)

**Figure 1:**
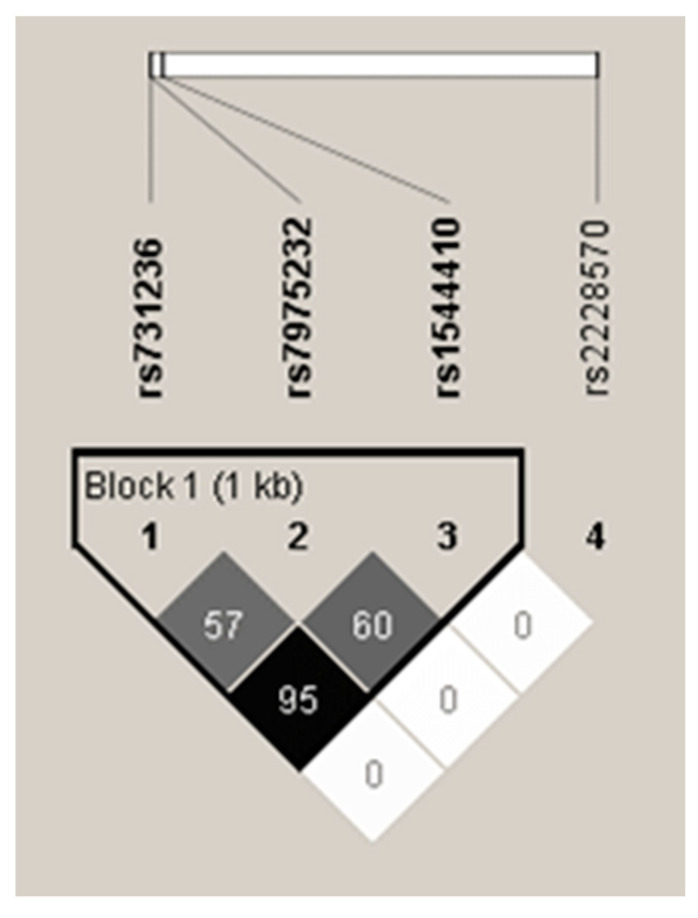
Values of linkage disequilibrium (LD) between the studied polymorphisms. Analysis was performed using Haploview 4.2 software.

*VDR* rs1544410, rs731236 and rs7975232 were found to be in strong linkage disequilibrium (LD) (Figure 1). Positions of rs731236 and rs7975232 are very close to each other in the gene. Additionally, all three mentioned polymorphisms are localized near the 3′ UTR of the gene. In contrast, locus of the rs2228570 SNP is in exon 1. Hence, it was not surprising that this one polymorphism was not in LD with the rest of the SNPs studied.

Allele frequencies obtained during the experiment respected the Hardy–Weinberg Equilibrium (HWE), with probability (*p*) values between 0.066 and 0.828.

### 3.2. Patients vs. Healthy Controls

No relations were found when comparing distribution of any of the studied *VDR* polymorphic variants between the axSpA patients and healthy controls. Data regarding this can be found in Table 2. The genotype and allele frequencies seen in the studied population closely resemble those reported in the NCBI SNP database.

**Figure 2:**
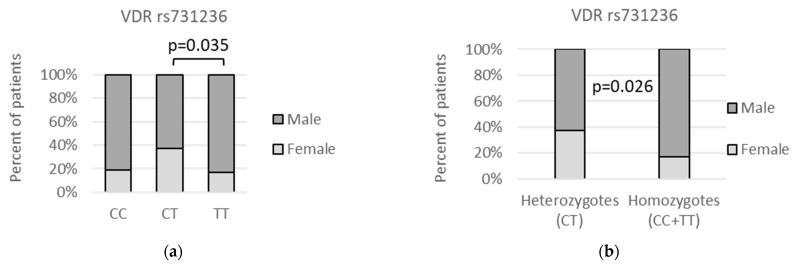
Relationship between *VDR* rs731236 genetic variants and patients’ gender. The following statistically significant associations were found: (**a**) *CT* vs. *TT*, *p* = 0.035, OR(95%CI) = 3 (1.16 to 7.56); and (**b**) *CC + TT* vs. *CT*, *p* = 0.026, OR(95%CI) = 0.35 (0.14 to 0.85). Analysis was performed using Fisher‘s exact test.

We have also found that the *VDR* rs731236 *CT* genotype was far more common among female (F) than male (M) patients in both codominant (*CT* vs. *TT*: (18/48 (37.5%) vs. 7/42 (16.7%); *p* = 0.035) and over-dominant (*CC* + *TT* vs. *CT*: 10/58 (17.2%) vs. 18/48 (37.5%); *p* = 0.026) models, as presented on Figure 2. Sadly, we were unable to perform similar analysis for the control group, as the sex of the healthy transplant donors has not been revealed to us.

### 3.3. VDR Polymorphisms and Patients’ Characteristics

No correlation was observed between the age of patient at diagnosis or the form of the disease and the presence of any of the studied polymorphic variants.

**Figure 3:**
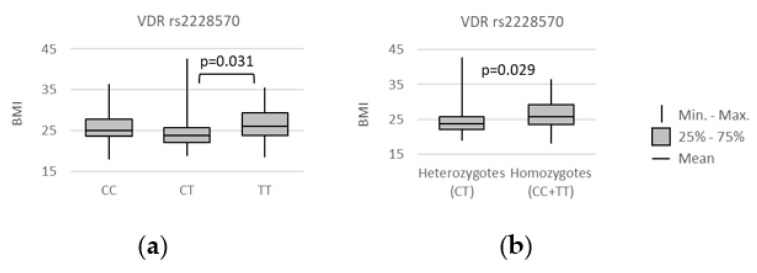
Relationships between *VDR* rs2228570 genotypes and patients’ body mass index (BMI), measured in kg/m^2^. The following statistically significant associations were found: (**a**) *CT* vs. *TT*, *p* = 0.031; (**b**) *CC + TT* vs. *CT*, *p* = 0.029. Analysis was performed using unpaired two-sample Wilcoxon test.

In turn, we found that body mass index (BMI) was lower in individuals carrying the rs2228570 *CT* genotype. The statistically significant associations were observed in the codominant (*CT* vs. *TT*: median(IQR) = 23.80 (22.05–25.89) vs. 26.08 (23.57–29.50); *p* = 0.031) and over-dominant (*CC + TT* vs. *CT*: median(IQR) = 25.85 (23.51–29.24) vs. 23.80 (22.05–25.89); *p* = 0.029) models, as shown on Figure 3. 

No associations were found between the studied *VDR* SNPs and presence of HLA-B27.

### 3.4. VDR polymorphisms and clinical parameters

No correlation was observed between CRP levels and any of the *VDR* polymorphic variants at any studied time point. However, we assessed a change in the parameters studied as an improvement equal to or higher than 50% and 75%, and in the case of BASDAI also as absolute values of ≤3 and ≤2. 

**Figure 4:**
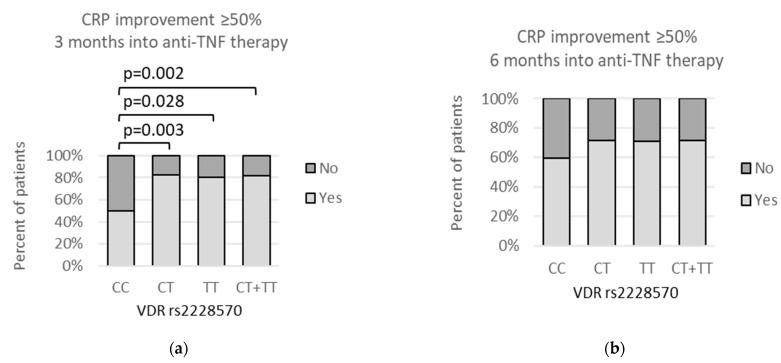
Relationships between *VDR* rs2228570 genetic variants and percent of patients achieving CRP improvement of ≥50% after (**a**) 3 months of anti-TNF therapy and (**b**) 6 months after beginning of the treatment. The following statistically significant associations were found: (**a**) *CC* vs. *CT*, *p* = 0.003, OR(95%CI) = 0.21 (0.08 to 0.59); *CC* vs. *TT*, *p* = 0.028, OR(95%CI) = 0.25 (0.08 to 0.81); and *CC* vs. *CT* + *TT*, *p* = 0.002, OR(95%CI) = 0.22 (0.09 to 0.59). Analysis was performed using Fisher‘s exact test.

We observed that an improvement equal to or higher than 50% in CRP after 3 months of anti-TNF therapy was associated with *VDR* rs2228570 genetic variants (Figure 4a). Lack of CRP improvement ≥50% after 3 months of anti-TNF therapy was related to the presence of the *CC* genotype. It was shown by dominant (*CC* vs. *CT* + *TT*: 16/32 (50%) vs. 13/71 (18.3%); *p* = 0.002) and codominant (*CC* vs. *CT*: 16/32 (50%) vs. 8/46 (17.4%); *p* = 0.003; *CC* vs. *TT*: 16/32 (50%) vs. 5/25 (20%); *p* = 0.028) models.

**Figure 5:**
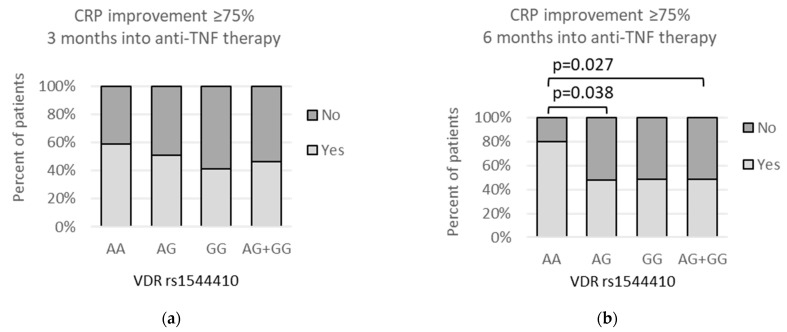
Relationships between *VDR* rs1544410 polymorphism and percent of patients achieving CRP improvement of ≥75% after (**a**) 3 months of anti-TNF therapy, and (**b**) 6 months after beginning of the treatment. The following statistically significant associations were found: (**b**) *AA* vs. *AG*, *p* = 0.038, OR(95%CI) = 4.36 (1.06 to 15.7); and *AA* vs. *AG + GG*, *p* = 0.027, OR(95%CI) = 4.29 (1.20 to 14.8);. Analysis was performed using Fisher‘s exact test.

As for the time point of 6 months after beginning of the treatment, the rs1544410 *AA* genotype was statistically more often observed among individuals who achieved CRP improvement equal to or higher than 75%, as shown by the dominant (*AA* vs. *AG* + *GG*: 12/15 (80%) vs. 42/87 (48.3%); *p* = 0.027) and the codominant (*AA* vs. *AG*: 12/15 (80%) vs. 22/46 (47.8%); *p* = 0.038) model (Figure 5b).

**Figure 6:**
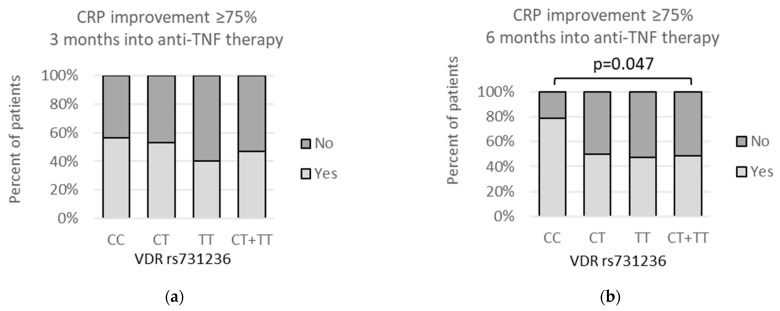
Relationships between *VDR* rs731236 genetic variants and percent of patients achieving CRP improvement ≥75% after (**a**) 3 months of anti-TNF therapy and (**b**) 6 months after beginning of the treatment. The following statistically significant associations were found: (**b**) *CC* vs. *CT + TT*, *p* = 0.047, OR(95%CI) = 3.84 (1.03 to 13.4). Analysis was performed using Fisher‘s exact test.

Similar observation was also made for the *VDR* rs731236 polymorphism. Here, 6 months after beginning anti-TNF therapy, the rs731236 *CC* genotype was related to CRP improvement equal to or greater than 75%, as compared to the *T* allele (11/14 (78.6%) vs. 43/88 (48.9%); *p* = 0.047) (Figure 6b).

**Figure 7:**
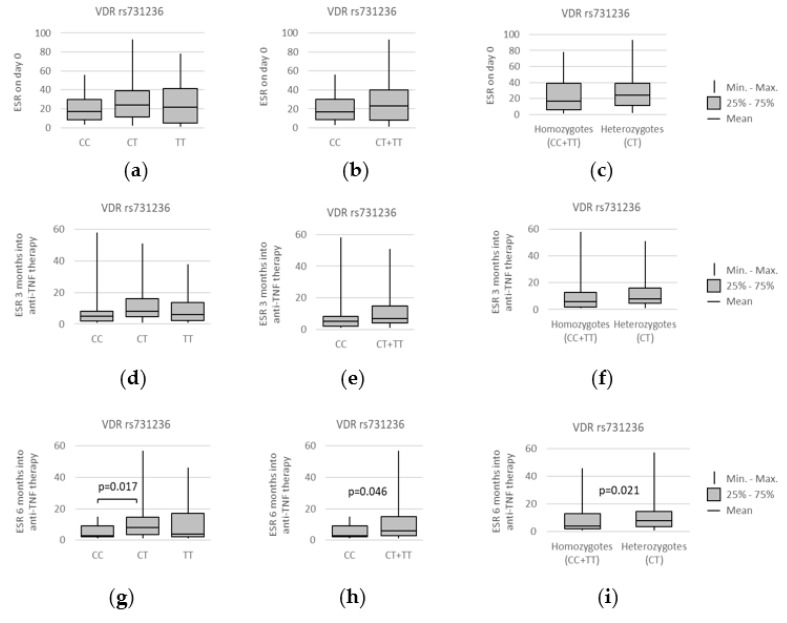
Relationships between *VDR* rs731236 genotypes and ESR levels at three time points: (**a**–**c**) prior to anti-TNF therapy, (**d**–**f**) after 3 months, and (**g**–**i**) after 6 months of the treatment. The following statistically significant associations were found: (**g**) *CC* vs. *CT*, *p* = 0.017; (**h**) *CC* vs. *CT + TT*, *p* = 0.046; and (**i**) *CC + TT* vs. *CT*, *p* = 0.021. Analysis was performed using unpaired two-sample Wilcoxon test.

Although erythrocyte sedimentation rate (ESR) measurement data was collected at all three time points: at baseline, and then 3 and 6 months after beginning of the anti-TNF treatment, the relationships with any of the *VDR* polymorphic variants were found to be statistically significant at only one time point. Namely, the lowest ESR after 6 months of therapy was observed for the rs731236 *CC* homozygotes in the codominant model (*CC* vs. *CT*: median (IQR) = 3 (2–11) vs. 8 (3–15); *p* = 0.017) as well as dominant model (*CC* vs. *CT* + *TT*: median (IQR) = 3 (2–11) vs. 6 (3–15); *p* = 0.046). Relationships between *VDR* rs731236 genotypes and ESR levels at different time points of the treatment are shown on Figure 7.

**Figure 8:**
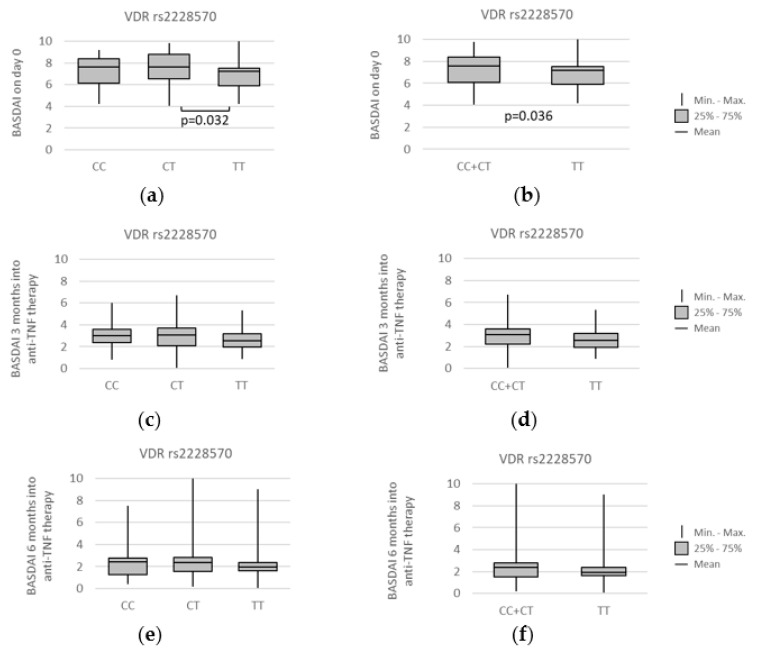
Relationships between *VDR* rs2228570 genotypes and BASDAI scores at different time points: (**a**,**b**) prior to anti-TNF therapy, (**c**,**d**) after 3 months, and (**e**,**f**) after 6 months of the treatment. The following statistically significant associations were found at day 0: (**a**) *CT* vs. *TT*, *p* = 0.032; and (**b**) *CC + CT* vs. *TT*, *p* = 0.036. Analysis was performed using unpaired two-sample Wilcoxon test.

Further statistical analysis revealed some noteworthy relations between the Bath Ankylosing Spondylitis Disease Activity Index (BASDAI) and the studied *VDR* polymorphisms. The first one worthy of mentioning was concerning the *VDR* rs2228570 polymorphism. Namely, median BASDAI before anti-TNF therapy was lower for individuals with the *TT* genotype, under the codominant model (*CT* vs. *TT*: median (IQR) = 7.65 (6.38–8.8) vs. 7.20 (5.55–7.5); *p* = 0.032) and the recessive model (*CC + CT* vs. *TT*: median (IQR) = 7.60 (6.1–8.4) vs. 7.20 (5.55–7.5); *p* = 0.036). Relationships between *VDR* rs2228570 genotypes and BASDAI at different time points of the treatment, as well as all statistically significant associations found, are presented on Figure 8.

**Figure 9:**
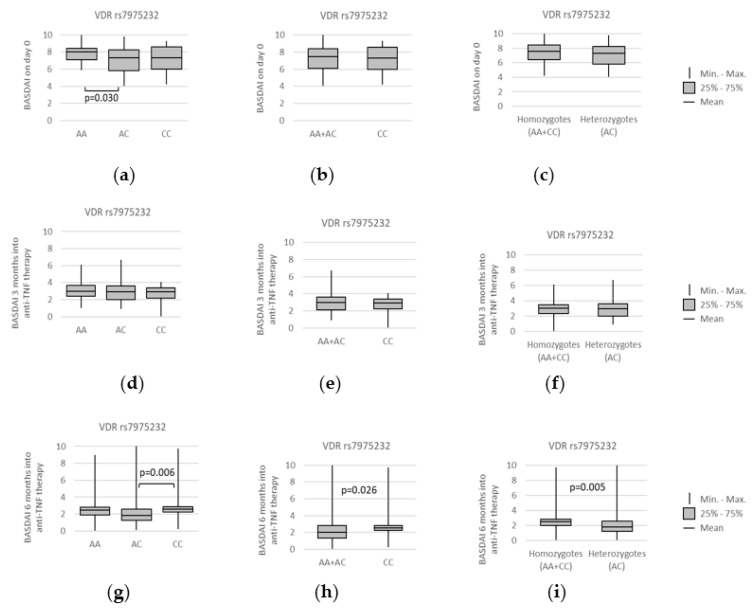
Relationships between and BASDAI scores and *VDR* rs7975232 genotypes at different time points: (**a**–**c**) prior to anti-TNF therapy, (**d**–**f**) after 3 months, and (**g**–**i**) after 6 months of the treatment. The following statistically significant association was found at day 0: (**a**) *AA* vs. *AC*, *p* = 0.030. Meanwhile, after 6 months into the treatment, the below relations were found: (**g**) *AC* vs. *CC*, *p* = 0.006; (**h**) *AA + AC* vs. *CC*, *p* = 0.026; and (**i**) *AA* + *CC* vs. *AC*, *p* = 0.005. Analysis was performed using unpaired two-sample Wilcoxon test.

The baseline BASDAI values also varied between distinct rs7975232 genotypes (Figure 9). Lower median BASDAI was observed in *AC* heterozygotes when compared to *AA* homozygotes (*AA* vs. *AC*, *p* = 0.030) (Figure 9a). 

All the above-mentioned relationships have not been preserved over time, and none of the studied VDR polymorphisms seemed to relate to BASDAI when 3 months have passed. However, this changed 6 months after the beginning of the anti-TNF treatment. At this time point, median BASDAI values were lower for rs7975232 *AC* heterozygotes under the over-dominant (*AA + CC* vs. *AC*: median (IQR) = 2.50 (1.95–2.85) vs. 1.80 (1.17–2.65); *p* = 0.005), as well as the codominant model (*AC* vs. *CC*: median (IQR) = 1.80 (1.17–2.65) vs. 2.55 (2.18–2.88); *p* = 0.006). The graphical representation of those associations can be found on Figure 9g,i.

**Figure 10:**
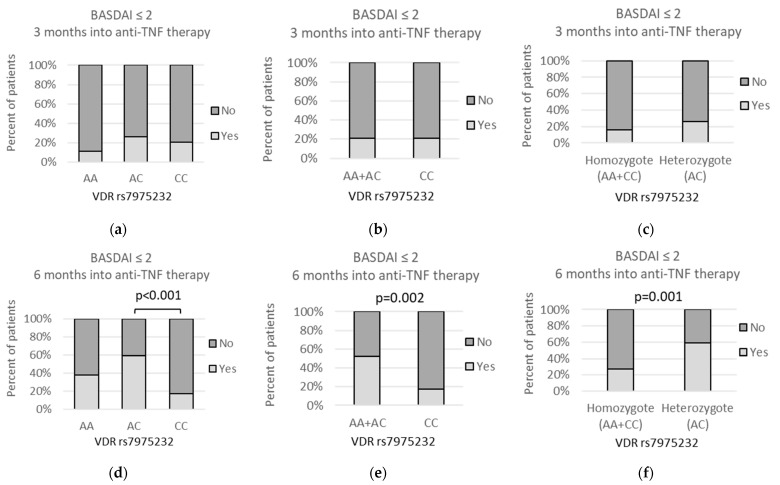
Relationships between *VDR* rs7975232 genetic variants and percent of patients achieving remission (BASDAI equal to or lower than 2 after (**a**–**c**) 3 months and (**d**–**f**) 6 months into the anti-TNF treatment. Prior to the therapy, BASDAI scores were above 4 for all the patients; therefore, this time point was not included in the current analysis. The following statistically significant associations were found after 6 months of the therapy: (**d**) *AC* vs. *CC*, *p* < 0.001, OR(95%CI) = 6.96 (2.30 to 18.52); (**e**) *AA + AC* vs. *CC*, *p* = 0.002, OR(95%CI) = 5.20 (1.75 to 13.4); and (**f**) *AA + CC* vs. *AC*, *p* = 0.001, OR(95%CI) = 0.26 (0.11 to 0.60). Analysis was performed using Fisher‘s exact test.

Similarly, we observed that after 6 months of anti-TNF treatment the rs7975232 *AC* genotype was more frequently seen in patients with remission: with BASDAI equal to or lower than 2 (Figure 10). The statistically significant associations were observed within over-dominant model (*AA + CC* vs. *AC*: 15/55 (27.3%) vs. 29/49 (59.2%); *p* = 0.001) and codominant model (*AC* vs. *CC*: 29/49 (59.2%) vs. 5/29 (17.2%); *p* < 0.001). Additionally, the presence of the *A* allele was more common in individuals having BASDAI of 2 or less under the recessive model (*AA* + *AC* vs. *CC*: 39/75 (52%) vs. 5/29 (17.2%); *p* = 0.002). Importantly, no such observation was made after 3 months of the therapy or prior to anti-TNF treatment (when the BASDAI scores were high and above 4 for all patients).

**Figure 11:**
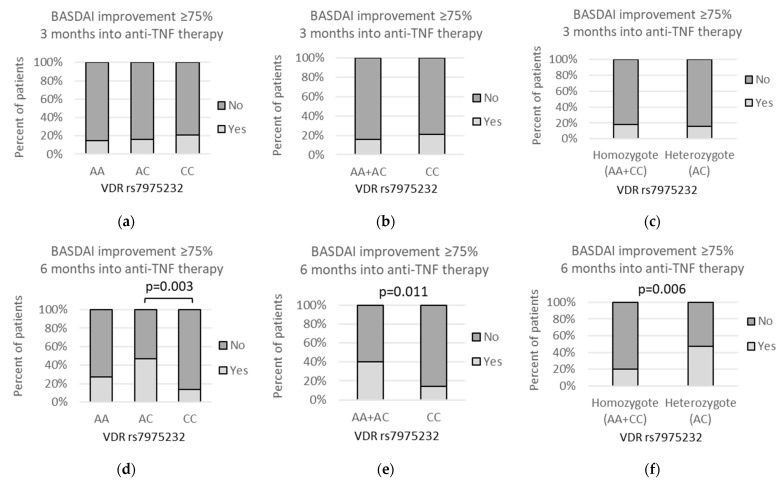
Relationships between *VDR* rs7975232 genetic variants and percent of patients with BASDAI improvement ≥75% after (**a**–**c**) 3 months and (**d**–**f**) 6 months into the anti-TNF treatment. The following statistically significant associations were found after 6 months of the therapy: (**d**) *AC* vs. *CC*, *p* = 0.003, OR(95%CI) = 5.53 (1.67 to 16.2); (**e**) *AA + AC* vs. *CC*, *p* = 0.011, OR(95%CI) = 4.17 (1.40 to 11.9); and (**f**) *AA + CC* vs. *AC*, *p* = 0.006, OR(95%CI) = 0.28 (0.12 to 0.65). Analysis was performed using Fisher‘s exact test.

The *VDR* rs7975232 *A* allele was further related to improvement in BASDAI after 6 months of anti-TNF treatment under the recessive model (*AA* + *AC* vs. *CC*: 30/75 (40%) vs. 4/29 (13.8%); *p* = 0.011). Furthermore, the over-dominant model (*AA + CC* vs. *AC*: 11/55 (20%) vs. 23/49 (46.9%); *p* = 0.006) and codominant model (*AC* vs. *CC*: 23/49 (46.9%) vs. 4/29 (13.8%); *p* = 0.003) showed that *AC* genotype was related to BASDAI improvement of ≥75%. The above-described associations are depicted on Figure 11.

It is of note that the statistical analysis did not reveal any significant correlations between the visual analogue scale (VAS) score and the presence of any of the *VDR* polymorphic variants. This did not change regardless of the time point studied.

Main results of the performed analysis concerning CRP, ESR, VAS, and BASDAI, including those that turned out to not be statistically significant, are shown in Supplementary Tables. 

### 3.5. VDR Polymorphisms and Other Symptoms

None of the *VDR* polymorphic variants analyzed in the experiment were found to be related with extra-articular manifestations, such as uveitis, enthesitis, psoriasis, or inflammatory bowel disease (IBD).

## 4. Discussion

Axial spondyloarthritis (axSpA) is a chronic inflammatory disease that mainly affects the axial skeleton. The term axSpA covers both patients who have already developed structural damage in the sacroiliac joints or spine visible on radiographs (radiographic axSpA, also termed ankylosing spondylitis (AS) and patients without such structural damage, labelled as non-radiographic axSpA [31]. The disease typically starts in young people, average age of onset ranges from 25 to 28 years. Diagnosis of the disease is still not ideal, with the mean diagnostic delay ranged from approximately 4 to 12 years. In addition to physical pain, the delay in treatment also leads to psychological suffering, such as distress or depression [32]. Treatment of axSpA also needs optimization and improvements. Up to 40% of patients treated with biological disease-modifying antirheumatic drugs (bDMARDs: tumor necrosis factor α inhibitors (TNFi) and the monoclonal antibodies against interleukin (IL)-17A) experience failure [33]. These aspects highlight the need for further research on axSpA.

Patients with AS, as in other autoimmune diseases, appear to have lower vitamin D levels than healthy controls; however, the cause is unclear. There emerged some suggestions in the literature that systemic inflammation may lower circulating levels of vitamin D [7].

In experimental models, 1,25(OH)2D downregulates the immune responses mediated by Th-1 cells, thus inhibiting the production of pro-inflammatory cytokines, such as Interferon-γ (IFN-γ), IL-2, IL-6, IL-17A, IL-17F, IL-22, IL-23, and TNF-α [34,35]. On the contrary, Th-2 cytokines, such as IL-4, IL-5, and anti-inflammatory cytokines IL-10 and transforming growth factor β (TGFβ), are promoted [34,36]. As a result, the shift of an inflammatory to a more tolerant immune status is promoted, which may explain the protective effects of vitamin D against autoimmune diseases [37]. However, despite the beneficial effects of 1,25(OH)2D supplementation in experimental autoimmune models, the application of vitamin D derivatives in clinical practice is currently limited to topical use for the treatment of psoriasis. The systemic use of vitamin D in the treatment of other autoimmune diseases is still under investigation [38].

Data from the literature on the effect of vitamin D receptor (*VDR*) gene polymorphisms on axSpA patients are small and mainly concern the incidence of AS. That is why in our current study we aimed to assess the relationship of four *VDR* polymorphisms: rs2228570 (*Fok*I), rs1544410 (*Bsm*I), rs7975232 (*Apa*I), and rs731236 (*Taq*I) with axSpA incidence, activity, treatment efficacy, and extra-articular symptoms in the Caucasian population. Even though no associations were identified between any of the studied polymorphisms and disease susceptibility, we found higher prevalence for the rs731236 *CT* genotype among female patients compared to males. Sex hormones influence the course of autoimmune diseases and SpAs is known to occur more frequently in men. In axSpA male sex is associated with greater spinal radiographic progression. Men are also more likely to have greater syndesmophyte formation. On the contrary, women tend to have greater enthesitis and experience more dactylitis [39]. In patients with AS, the different activation of the immune system, particularly Th17 axis, supports a distinct sexual dimorphism since males but not females show higher frequency of IL-17A and Th17 cells [40]. Estrogen-mediated effects on immune response may favor a Th1 profile or a Th2 profile, depending on hormone concentration. Moreover, estrogen has been demonstrated to enhance vitamin D function favoring its accumulation and increasing the expression of *VDR*. This may result in a more potent anti-inflammatory response in females than males. On the contrary, vitamin D has been shown to downregulate the expression of aromatase in immune cells, which converts testosterone to estrogen, leading to a decrease in estrogen level [41].

Pimenta et al. [42], who also studied Caucasians, similarly did not find any correlations with axSpA susceptibility and of the *VDR* gene polymorphisms: rs2228570, rs731236, and rs7975232. In turn, in southern Brazil—where the population is classified as mixed ethnicity with predominantly European origin—a group of SpA patients with AS and psoriatic arthritis was evaluated [43]. The rs7975232 *CC* and the rs2228570 *CC* genotypes were a risk factor for SpA and AS in males. Additionally, the rs731236 *CC* genotype and the rs7975232 *AC* genotype were important risk factors for developing lumbar spine pathologies in the general population [44].

As for other populations, group of Cai et al. [45] assessed the incidence of AS in a large number of individuals of Chinese origin. Among the four polymorphisms studied (rs1544410, rs2228570, rs731236, and rs7975232), the rs731236 *G* allele was shown to be statistically more frequent in AS patients in comparison to healthy controls. In addition, a correlation with AS morbidity was noted for the haplotypes rs731236 and rs1544410. Haplotypes *AC* and *GT* were found to play protective and risk roles, respectively [45]. However, the data were not confirmed in a study of the Asian population by Zhang et al. [46]. In that latter study, the only statistically significant relationship concerned the rs11168266-rs11168267 haplotype *TG* and disease susceptibility.

The data regarding the correlation of vitamin D levels with ESR, CRP, and BASDAI scores are conflicting [7]. In the study presented here, we found some correlations of *VDR* polymorphisms with disease activity and treatment efficacy. In particular, the baseline BASDAI values were lower in rs2228570 *TT* homozygotes compared to the *C* allele carriers (*p* = 0.036). Furthermore, a lower BASDAI score was observed in individuals with the rs7975232 *C* allele in comparison to the *AA* homozygotes (*p* = 0.029). We also found that the rs7975232 genetic variants were related to BASDAI values after 6 months of anti-TNF treatment.

Pimenta et al. [42] assessed axSpA patients using BASDAI, Bath Ankylosing Spondylitis Functional Index (BASFI), Bath Ankylosing Spondylitis Metrology Index (BASMI), muscle physical properties (stiffness, tone, elasticity), muscle strength, muscle mass, and muscle performance measured by 60 s sit-to-stand test (ST60) and gait speed. The only significant correlation that they found concerned *VDR* rs2228570 and reduced muscle performance measured by gait speed in the group of patients compared to controls.

Another team of scientists [47] described an association between *VDR* rs2228570 SNP and bone mineral density in AS patients. Here, higher CRP values were described for the rs2228570 *CC* genotype. The authors also mentioned a correlation with ESR levels but did not indicate the relevant genotype. Inflammatory activity was explained by a reduction in TNF-α mRNA and protein expression in vitro.

Regarding mentioned indicators of inflammation, our current study showed an improvement in CRP after 3 months of anti-TNF therapy in rs2228570 *T* allele carriers compared to the *CC* homozygotes (*p* = 0.002). When 6 months of the treatment passed, CRP improvement was related to the rs1544410 *AA* genotype in comparison to the *G* allele (*p* = 0.027), as well as with the rs731236 *CC* genotype compared to the *T* allele carriers (*p* = 0.047).

For other inflammatory joint disease–rheumatoid arthritis (RA)–the *GG* genotype of the *VDR* rs1544410 polymorphism was found to be associated with less severe disease in one study [14], and the second study highlighted higher activity for the *GG* and the *AG* rs1544410 genotypes [48].

Rs2228570 polymorphism was associated with better clinical activity measured by BASDAI score in PsA patients [43]. Published data on axSpA treatment efficacy related to polymorphisms of other genes but not *VDR*.

Biological effects of proinflammatory TNF include activation of other cells (macrophages, T-cells, B-cells), inflammatory cytokine production (IL-1, IL-6), expression of adhesion molecule (ICAM-1, E-selectin), inhibition of regulatory T-cells, matrix metalloproteinase production and induction of apoptosis [49]. An interesting effect of vitamin D was described by Dankers et al. [38]. Adding increasing dosages of 1,25(OH)2D augments the effects of anti-TNF etanercept. Combining 1,25(OH)2D and dexamethasone with etanercept has additive effects compared with etanercept alone. Binding and neutralizing activities against soluble TNF-α (sTNF) are the critical and common mechanisms of action of anti-TNF agents. On the contrary, studies have demonstrated that these agents have additional biological effects against precursor form transmembrane TNF-α (tmTNF)-and Fc receptor-expressing cells [50]. Vitamin D appears to have an anti-inflammatory effect, as do anti-TNF drugs. In a cytokine-dependent network, vitamin D receptor polymorphisms may enhance the anti-inflammatory properties of the drugs, as reflected in the treatment efficacy described in our study.

Lastly, the limitations of our current study should be noted. Genetic studies require a large number of patients: 100 individuals may not be enough to find some polymorphisms’ relationships, especially those that occur rarely. In order to increase the study group, we counted subjects with AS and non-radiographic axSpA together. In our study, Caucasian people were described; therefore, the results may differ according to ethnic differences. New insights into the genetics of axSpA were possible by analysis of disease activity based on clinical as well as laboratory features. Several examinations during treatment follow-up allowed us to analyze anti-TNF treatment effectiveness, which is a new perspective in axSpA genetics research.

## 5. Conclusions

*VDR* SNPs were found to relate to CRP and BASDAI values at different time points of anti-TNF therapy.

## Figures and Tables

**Table 1:** Characteristics of the patient cohort included in the study. SD, standard deviation; F, female; M, male; nr-axSpA, non-radiographic axial spondyloarthritis; AS, ankylosing spondylitis; BMI, body mass index; HLA-B27, human leukocyte antigen B27; MTX, methotrexate; NSAIDs, nonsteroidal anti-inflammatory drugs; BASDAI, Bath Ankylosing Spondylitis Disease Activity Index.

Number of Patients (N)	106
Age mean in years (±SD)	42.7 (±12.9)
Disease duration mean in years (±SD)	9.29 (±8.49)
Disease onset mean in years (±SD)	32.7 (±10.2)
Sex F/M (%)	28/78 (73.6%)
BMI mean (±SD)	25.5 (±4.59)
HLA-B27 positive patients, %	88%
Form axial/axially peripheral (%)	58 (54.7%)/48 (45.3%)
nr-axSpA/AS (%)	20 (18.9%)/86 (81.1%)
**Extra-articular manifestations:**	**N (%)**
Uveitis	33 (31.1%)
Inflammatory bowel disease	18 (17.0%)
Enthesitis	17 (16.0%)
Psoriasis	6 (5.7%)
Patients with at least one manifestation	53 (50.0%)
Patients with two manifestations or more	17 (16.0%)
**Concomitant treatment at the start of biologic treatment:**	**N (%)**
NSAIDs	73 (69.5%)
MTX	32 (30.2%)
Sulfasalazine/Mesalazine	28 (26.4%)
Corticosteroids	17 (16.0%)
Other	2 (2.0%)
**Anti-TNF drugs:**	**N (%)**
Adalimumab	43 (40.6%)
Etanercept	28 (26.4%)
Certolizumab	24 (22.6%)
Golimumab	9 (8.5%)
Infliximab	2 (2.0%)
**Disease activity:**	
BASDAI before treatment, median (range)	7.45 (4.05–10)
BASDAI after 6 months of treatment, median (range)	2.30 (0–10)
Low activity ^1^ after 6 months of treatment, N (%)	97 (93.3%)

^1^ Defined as BASDAI < 3.

**Table 2:** Genotype and allele frequencies of studied VDR SNPs in patients with axial spondyloarthritis (axSpA) and in healthy controls.

**SNP/Genotype**	axSpA [**N (%)]**	**Healthy Controls [N (%)]**
rs1544410		
*AA*	17 (16.0%)	27 (22.1%)
*AG*	48 (45.3%)	52 (42.6%)
*GG*	41 (38.7%)	43 (35.3%)
*A*	82 (38.7%)	106 (43.4%)
*G*	130 (61.3%)	138 (56.6%)
rs2228570		
*CC*	33 (31.1%)	39 (32.0%)
*CT*	48 (45.3%)	63 (51.6%)
*TT*	25 (23.6%)	20 (16.4%)
*C*	114 (53.8%)	141 (57.8%)
*T*	98 (46.2%)	103 (42.2%)
rs731236		
*CC*	16 (15.1%)	15 (12.9%)
*CT*	48 (45.3%)	65 (56.1%)
*TT*	42 (39.6%)	36 (31.0%)
*C*	80 (37.7%)	95 (40.9%)
*T*	132 (62.3%)	137 (59.1%)
rs7975232		
*AA*	27 (25.5%)	26 (22.4%)
*AC*	50 (47.2%)	65 (56.0%)
*CC*	29 (27.3%)	25 (21.6%)
*A*	104 (49.1%)	117 (50.4%)
*C*	115 (50.9%)	115 (49.6%)

## Data Availability

The datasets generated and/or analyzed during the current study are available from the corresponding author on reasonable request.

## Supplementary Materials

The following supporting information can be downloaded at: https://www.mdpi.com/article/10.3390/genes13101873/s1, Table S1: rs1544410, Table S2: rs2228570, Table S3: rs731236, Table S4: rs7975232.
